# Red blood cell count and risk of adverse outcomes in patients with mildly reduced left ventricular ejection fraction

**DOI:** 10.1002/clc.24108

**Published:** 2023-08-04

**Authors:** Zhican Liu, Yunlong Zhu, Lingling Zhang, Mingxin Wu, Haobo Huang, Ke Peng, Wenjiao Zhao, Sihao Chen, Xin Peng, Na Li, Hui Zhang, Yuying Zhou, Yiqun Peng, Jie Fan, Jianping Zeng

**Affiliations:** ^1^ Department of Cardiology Xiangtan Central Hospital Xiangtan Hunan China; ^2^ Graduate Collaborative Training Base of Xiangtan Central Hospital, Hengyang Medical School University of South China Hengyang Hunan China; ^3^ Department of Scientific Research Xiangtan Central Hospital Xiangtan Hunan China

**Keywords:** anemia, heart failure, HFmrEF, RBC, red blood cell

## Abstract

**Background:**

Anemia is associated with increased rates of heart failure (HF)‐related mortality and hospitalization. No studies have focused on the association between the red blood cell (RBC) count and the prognosis of patients with HF with mildly reduced left ventricular ejection fraction (HFmrEF). We retrospectively analyzed the effect of the RBC count on outcome events in patients with HFmrEF.

**Methods:**

We investigated the association of the RBC count with outcome events in 1691 patients with HFmrEF (mean age: 68 years; 35% female) in Xiangtan Central Hospital. Using Cox proportional hazards models, the RBC count was assessed as both a continuous and categorical variable.

**Results:**

During follow‐up (median: 33 months), cardiovascular death occurred in 168 patients (114 men and 54 women). After adjusting for established risk factors, each 1.0 × 10^12^ cell/L increase in the RBC count was associated with a 28% lower risk of cardiovascular death in men and a 43% lower risk in women. Patients with low RBC counts had a 0.5‐fold higher risk of cardiovascular death than those with normal RBC counts. The hazard ratio for men was 1.42 (95% confidence interval [CI]: 1.07–1.89), and the hazard ratio for women was 1.79 (95% CI: 1.20–2.67). The RBC count was not significantly associated with the composite endpoint of cardiovascular death and HF readmission (cardiovascular events) (*p* > .05).

**Conclusions:**

A decreased RBC count is associated with increased cardiovascular mortality in patients with HFmrEF. Correcting a low RBC count might potentially reduce the risk of cardiovascular death in patients with HFmrEF.

## Graphical abstract

We investigated the association of the RBC count with outcome events in 1691 patients with HFmrEF (mean age: 68 years; 35% female). During follow‐up (median: 33 months), cardiovascular death occurred in 168 patients (114 men and 54 women). A decreased RBC count is associated with increased cardiovascular mortality in patients with HFmrEF. Each 1.0 × 10^12^ cell/L increase in the RBC count was associated with a 28% lower risk of cardiovascular death in men and a 43% lower risk in women.

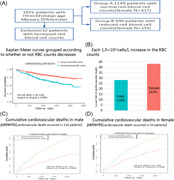

## 1 INTRODUCTION

Significant progress has been made in the pathogenesis of heart failure (HF), and reasonable treatment has improved patients’ prognoses.^1^ However, the prognosis of HF remains unsatisfactory.^2^, ^3^ Anemia is common in patients with HF,^3^ and the pathogenesis of anemia in HF is multifactorial.^4^, ^5^ Patients are classified as having HF with reduced ejection fraction [HFrEF; left ventricular ejection fraction (LVEF) of ≤40%], HF with mildly reduced ejection fraction (HFmrEF; LVEF of 41%–49%), or HF with preserved ejection fraction (HFpEF; LVEF of ≥50%).^1^ Furthermore, studies have shown that anemia is an independent predictor of mortality and morbidity in patients with HFrEF or HFpEF.^5^, ^6^ Few studies have examined the relationship between anemia and HFmrEF or between the red blood cell (RBC) count and HFmrEF; therefore, whether changes in the RBC count impact the prognosis of patients with HFmrEF remains unclear. In the present study, we retrospectively analyzed the effect of the RBC count on outcome events in patients with HFmrEF. At the same time, we conducted a stratified analysis of men and women with HFmrEF to determine whether changes in the RBC count result in differences in outcome events in men and women with HFmrEF. The aim of this study is to increase the overall understanding of the characteristics of patients with HFmrEF and formulate more reasonable treatment for these patients.

## 2 METHODS

### 2.1 Three critical parameters related to RBCs

RBC count: This denotes the concentration of RBCs per microliter of blood, typically enumerated in millions. Integral to oxygen transportation, diminished counts may indicate conditions such as anemia, while elevated counts could signal disorders like polycythemia vera.

Hemoglobin: This intracellular protein is responsible for transporting oxygen to tissues and returning carbon dioxide to the lungs. Hemoglobin concentrations, quantified in grams per deciliter, can reflect various health conditions, with lower levels potentially indicative of anemia and higher levels suggestive of polycythemia vera or chronic hypoxia.

Red blood cell specific volume (RBCSV): This parameter represents the volume fraction of RBCs in the blood, also called hematocrit. Normal hematocrit ranges differ by sex. Deviations from normal ranges can indicate various conditions—low levels may suggest anemia, and high levels could indicate dehydration or other disorders.

### 2.2 RBC count

According to the definition of the standard RBC count in routine blood testing at our hospital, the reference range for men is 4.0 to 5.5 × 10^12^/L and that for women is 3.5 to 5.0 × 10^12^/L. After excluding patients with elevated RBC counts (>5.5 × 10^12^/L in men and >5.0 × 10^12^/L in women), the patients were divided into those with reduced RBC counts (<4.0 × 10^12^/L in men and <3.5 × 10^12^/L in women) and normal RBC counts (≥4.0 × 10^12^/L in men and ≥3.5 × 10^12^/L in women). We chose to evaluate RBC count as our primary parameter rather than hemoglobin or RBC specific volume (hematocrit), because RBC count is less affected by hypoxia, dehydration, and volume status, which can be confounding clinical factors within this patient population.

### 2.3 Participants

**Figure 1:**
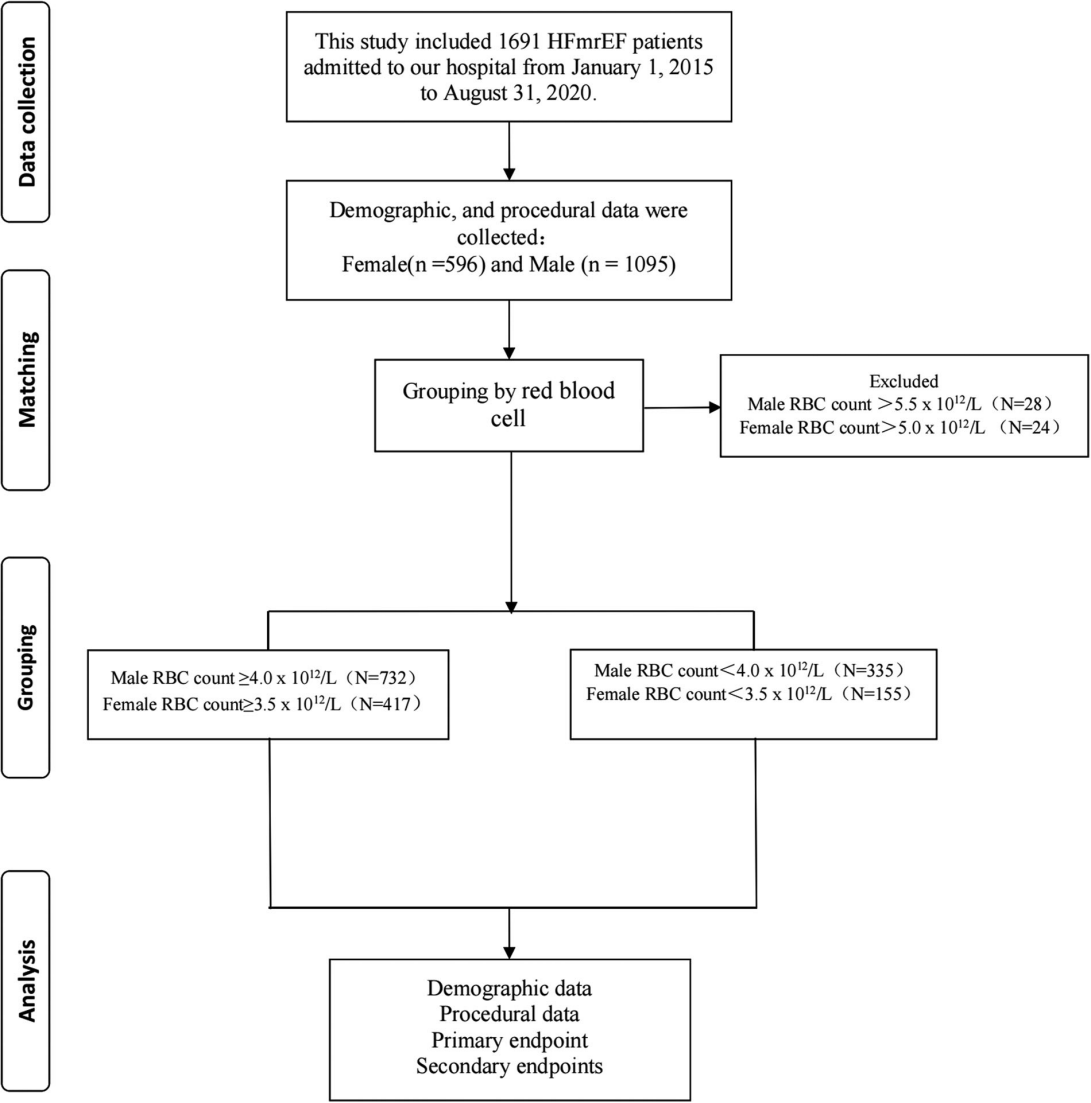
Flow diagram for participant screening, eligibility and analysis.

The study protocol was approved by the Ethics Committee of Xiangtan Central Hospital (Xiangtan, China) and conformed to the principles outlined in the Declaration of Helsinki.^7^ Informed consent was obtained from all patients or their guardians before the study protocol was initiated. The requirement for written informed consent was waived because of the study's retrospective nature; consent was only obtained verbally in person or by telephone. This study was based on the Outcome of Discharged HFmrEF Patients study (OUDI‐HF study; ClinicalTrials.gov number NCT05240118). The OUDI‐HF study included 1691 patients with HFmrEF who were admitted to our hospital from January 1, 2015 to August 31, 2020. The inclusion criteria were HF with an LVEF of 41%–49% and a New York Heart Association HF score of II–IV. The exclusion criteria were malignancies or other non‐cardiac diseases with expected survival of less than 1 year. After excluding 52 patients with increased RBC counts, 1149 patients with normal RBC counts and 490 patients with decreased RBC counts were included in the study (Figure 1).

### 2.4 Outcomes

Demographic and procedural data were collected from the patients’ hospital charts or the hospital database. All study participants were followed up on August 31, 2021. A panel of seven experienced physicians reviewed suspected cardiovascular (CV) events by examining the information obtained from hospital records and follow‐ups, including clinical telephone interviews and community visits. The primary outcome of interest was CV death after discharge, and the secondary outcome was the composite of CV death and HF readmissions (CV events). CV death was defined as death from any CV mechanism, including acute myocardial infarction, sudden cardiac death, HF, stroke, CV surgery, CV hemorrhage, and other CV causes.

### 2.5 Statistical analysis

We used Cox proportional hazards regression models stratified by cohort to examine the association of the RBC count with the rate of outcome events. We performed a sex‐stratified analysis. The RBC count was assessed as a continuous variable (increased risk was calculated for each 1.0 × 10^12^ cell/L increase) and as a categorical variable. We adjusted for the following baseline covariates: age, body mass index，smoking status, alcohol use, hypertension, hyperlipidemia, diabetes, coronary heart disease, atrial fibrillation, previous stroke, chronic obstructive pulmonary disease, renal insufficiency, New York Heart Association functional class, myocardial infarction, percutaneous coronary intervention, uric acid concentration, estimated glomerular filtration rate, potassium concentration and LVEF.

The credibility of the Cox proportional risk regression model was verified using propensity score matching analysis. Propensity score matching analysis based on a multivariate logistic regression model with the following factors: age, body mass index, gender, smoking status, alcohol use, hypertension, hyperlipidemia, diabetes, coronary heart disease, atrial fibrillation, previous stroke, chronic obstructive pulmonary disease, renal insufficiency, New York Heart Association functional class, myocardial infarction, percutaneous coronary intervention, uric acid concentration, estimated glomerular filtration rate, potassium concentration and LVEF. Propensity scores were calculated by the dichotomous variable of whether the patient's RBC count was reduced, and then the scores were matched 1:1, allowing for a difference in scores of 0.01. This strategy results in 389 matching pairs per group.

Clinical characteristics were compared between the groups using the *t‐*test for continuous variables and the Chi‐square test for categorical variables. The Kaplan–Meier method was used to estimate the incidence of cumulative events. All *p* values were obtained using the Kruskal–Wallis rank‐sum test for continuous variables and Fisher's exact probability test for count variables. The results were considered significant when the *p* value was <.05. All analyses were performed using R (http://www.R-project.org) and EmpowerStats (www.empowerstats.net, X&Y Solutions, Inc.).

## 3 RESULTS

### 3.1 Baseline characteristics

More than half of the 1639 patients included in this study were male (*n* = 1067). The patients’ mean age was 68.5 ± 12.3 years, their mean body mass index was 25.1 ± 4.1 kg/m^2^, their mean LVEF was 44.4% ± 2.7%, and more than half of the patients had been hospitalized for myocardial infarction (51.8%) (Table 1).

**Table 1:** Baseline characteristics according to whether the RBC count decreases (*n* = 1639).

	Male			Female		
	RBC count ≥4.0 × 10^12^/L	RBC count <4.0 × 10^12^/L	*p* value	RBC count ≥3.5 × 10^12^/L	RBC count <3.5 × 10^12^/L	*p* value
No	*N* = 732	*N* = 335		*N* = 417	*N* = 155	
Age, years	66.0 ± 12.2	71.5 ± 12.3	**<.001**	70.6 ± 11.4	68.2 ± 12.6	**.036**
Body mass index, kg/m^2^	25.4 ± 4.0	24.6 ± 4.0	**.001**	25.1 ± 4.2	24.6 ± 4.2	.161
Current smoker, *N* (%)	359 (49.0%)	128 (38.2%)	**<.001**	33 (7.9%)	11 (7.1%)	.745
Current drinker, *N* (%)	110 (15.0%)	26 (7.8%)	**<.001**	6 (1.4%)	1 (0.6%)	.443
Medical history, *N* (%)						
Coronary heart disease	589 (80.5%)	264 (78.8%)	.530	320 (76.7%)	113 (72.9%)	.342
Hypertension	467 (63.8%)	250 (74.6%)	**<.001**	295 (70.7%)	118 (76.1%)	.201
Hyperlipidemia	158 (21.6%)	45 (13.4%)	**.002**	101 (24.2%)	33 (21.3%)	.462
Atrial fibrillation	132 (18.0%)	48 (14.3%)	.134	76 (18.2%)	26 (16.8%)	.687
Diabetes	212 (29.0%)	124 (37.0%)	**.009**	135 (32.4%)	65 (41.9%)	**.033**
Previous stroke	88 (12.0%)	42 (12.5%)	.811	57 (13.7%)	18 (11.6%)	.517
Myocardial infarction	436 (59.6%)	158 (47.2%)	**<.001**	198 (47.5%)	57 (36.8%)	**.022**
PCI	311 (42.5%)	86 (25.7%)	**<.001**	128 (29.0%)	30 (19.4%)	**.019**
COPD	110 (15.0%)	65 (19.4%)	.073	21 (5.0%)	10 (6.5%)	.506
Renal insufficiency	129 (17.6%)	136 (40.6%)	**<.001**	65 (15.6%)	73 (47.1%)	**<.001**
Clinical profile						
NYHA III + IV, *N* (%)	380 (51.9%)	206 (61.5%)	**.008**	249 (59.7%)	109 (70.3%)	**.005**
shortness of breath, *N* (%)	297 (40.6%)	182 (54.3%)	**<.001**	198 (47.5%)	95 (61.3%)	**.003**
Jugular venous distention, *N* (%)	65 (8.9%)	40 (11.9%)	.119	49 (11.8%)	31 (20.0%)	**.011**
Leg edema, *N* (%)	118 (16.1%)	119 (35.5%)	**<.001**	93 (22.3%)	71 (45.8%)	**<.001**
Systolic BP, mmHg	134.2 ± 24.7	139.0 ± 27.1	**.005**	135.6 ± 24.4	142.0 ± 30.3	**.009**
Heart rate, bpm	82.6 ± 19.8	84.2 ± 17.8	.204	84.5 ± 20.6	87.7 ± 19.5	.089
Medications, *N* (%)						
Calcium channel blocker	257 (35.1%)	146 (43.6%)	**.008**	158 (37.9%)	81 (52.3%)	**.002**
Beta‐blocker	603 (82.4%)	247 (73.7%)	**.001**	335 (80.3%)	123 (79.4%)	.794
ACEi	428 (58.5%)	138 (41.2%)	**<.001**	217 (52.0%)	51 (32.9%)	**<.001**
ARB	190 (26.0%)	78 (23.3%)	.350	128 (30.7%)	43 (27.7%)	.493
ARNI	34 (4.6%)	18 (5.4%)	.608	18 (4.3%)	6 (3.9%)	.813
Loop diuretics	346 (47.3%)	202 (60.3%)	**<.001**	234 (56.1%)	89 (57.4%)	.780
Spironolactone	354 (48.4%)	143 (42.7%)	.085	201 (48.2%)	51 (32.9%)	**.001**
Laboratory findings						
Hemoglobin, g/dL	137.7 ± 13.2	101.1 ± 19.3	**<.001**	120.8 ± 12.6	89.6 ± 15.2	**<.001**
red blood cell count, 10^12^/L	4.6 ± 0.4	3.4 ± 0.5	**<.001**	4.1 ± 0.4	3.0 ± 0.4	**<.001**
Red blood cell specific volume, %	42.3 ± 4.4	31.6 ± 5.6	**<.001**	37.9 ± 4.7	28.7 ± 6.1	**<.001**
Uric acid, µmol/L	368.0 ± 112.7	387.9 ± 123.3	**.009**	331.7 ± 106.8	377.7 ± 131.1	**<.001**
eGFR, mL/min/1.73 m^2^	79.7 ± 30.0	55.0 ± 37.5	**<.001**	71.7 ± 34.1	43.1 ± 35.1	**<.001**
Sodium, mmol/L	139.4 ± 3.5	139.5 ± 3.8	.618	139.3 ± 3.5	139.0 ± 3.9	.252
Potassium, mmol/L	4.1 ± 0.5	4.3 ± 0.7	**<.001**	4.1 ± 0.6	4.4 ± 0.9	**<.001**
Echocardiography						
LAs, mm	38.8 ± 6.1	40.4 ± 5.9	**<.001**	39.0 ± 6.5	39.8 ± 5.4	.136
LVd, mm	54.4 ± 6.5	55.8 ± 7.6	**.001**	52.5 ± 7.0	52.8 ± 6.7	.554
RAs, mm	37.8 ± 6.2	38.5 ± 6.1	.057	36.8 ± 6.2	36.5 ± 5.4	.576
RVd mm	20.8 ± 4.9	21.9 ± 6.2	**.002**	20.2 ± 5.0	20.4 ± 4.8	.665
LVEF, %	44.5 ± 2.7	44.5 ± 2.8	.914	44.4 ± 2.8	44.1 ± 2.7	.155

*Note*: *The population was classified according to whether the male RBC count was ≥4.0 × 10^12^/L and whether the female RBC count was ≥3.5 × 10^12^/L. Values for continuous variables are given as means ± *SD*. **Bold** represent significant values (*p* < .05).

Abbreviations: ACEi, angiotensin‐converting enzyme inhibitor; ARB, angiotensin receptor blocker; ARNI, angiotensin receptor neprilysin inhibitor; COPD, chronic obstructive pulmoriary disease; eGFR, estimated glomerular filtration rate; LAs, left atrial size; LVd, left ventricle dimension; LVEF, left ventricular ejection fraction; NYHA, New York Heart Association; PCI, percutaneous coronary intervention; RAs, right atrial size; RBC, red blood cell; RVd, right ventricle dimension.

The mean RBC count was 4.2 ± 0.7 × 10^12^/L in men and 3.8 ± 0.6 × 10^12^/L in women. Men with reduced RBC counts had higher rates of hypertension, diabetes, and renal insufficiency but lower rates of hyperlipidemia and myocardial infarction. Women with reduced RBC counts had higher rates of diabetes and renal insufficiency but lower rates of myocardial infarction (Table 1).

### 3.2 RBC count and risk of outcome events

During a median follow‐up of 33 months, 383 patients (242 men and 141 women) had CV deaths and 953 patients (618 men and 335 women) had CV events. A reduced RBC count was associated with a doubling of CV mortality compared with a normal RBC count (hazard ratio [HR]: 2.28; 95% confidence interval [95% CI]: 1.80–2.90; *p* < .0001]. The incidence of CV events in patients with reduced RBC counts was only associated with male sex (HR: 1.41; 95% CI: 1.08–1.84; *p* = .0114), not female sex (HR: 1.21; 95% CI: 0.83–1.77; *p* = .3190) (Table 2).

**Table 2:** Univariate analysis was performed on the cumulative incidence of outcome events according to RBC count grouping

	Male			Female			Total		
	Event, *N* (%)	Hazard ratio (95% CI)	*p* value	Event, *N* (%)	Hazard ratio (95% CI)	*p* value	Event, *N* (%)	Hazard ratio (95% CI)	*p* value
**Cardiovascular death**									
Male RBC count ≥4.0 × 10^12^/L or Female RBC count ≥3.5 × 10^12^/L	128 (17.49)	(Reference)		87 (20.86)	(Reference)		215 (18.71)	(Reference)	
Male RBC count <4.0 × 10^12^/L or Female RBC count <3.5 × 10^12^/L	114 (34.03)	2.43 (1.81, 3.27)	**<.0001**	54 (34.84)	2.03 (1.35, 3.04)	**.0006**	168 (34.29)	2.28 (1.80, 2.90)	**<.0001**
**Cardiovascular event (cardiovascular death and heart failure readmission)**									
Male RBC count ≥4.0 × 10^12^/L or Female RBC count ≥3.5 × 10^12^/L	405 (55.33)	(Reference)		239 (57.31)	(Reference)		644 (56.05)	(Reference)	
Male RBC count <4.0 × 10^12^/L or Female RBC count <3.5 × 10^12^/L	213 (63.58)	1.41 (1.08, 1.84)	**.0114**	96 (61.94)	1.21 (0.83, 1.77)	.3190	309 (63.06)	1.34 (1.08, 1.67)	**.0082**

*Note*: *The population was classified according to whether the male RBC count was ≥4.0 × 10^12^/L and whether the female RBC count was ≥3.5 × 10^12^/L. **Bold** represent significant values (*p* < .05).

Abbreviations: CI, confidence interval; RBC, red blood cell.

After adjusting for age and body mass index, each 1.0 × 10^12^ cell/L increase in the RBC count was associated with a 41% lower risk of overall CV death (HR: 0.59; 95% CI: 0.51–0.67; *p* < .0001). The risk of CV death was reduced by 49% in women (HR: 0.51; 95% CI: 0.40–0.67; *p* < .0001) and by 37% in men (HR: 0.63; 95% CI: 0.53–0.75; *p* < .0001) (Table 3, Model I[A]). Patients with reduced RBC counts had a 97% higher risk of CV death than patients with normal RBC counts (HR: 1.97; 95% CI: 1.61–2.42; *p* < .0001), including a 111% higher risk in women (HR: 2.11; 95% CI: 1.50–2.97; *p* < .0001) and 86% higher risk in men (HR: 1.86; 95% CI: 1.43–2.40; *p* < .0001) (Table 3, Model I[B]). The RBC count was not significantly correlated with the incidence of CV events (*p* > .05) (Table 3, Model III[A,B]).

**Table 3:** Results of a multivariate Cox proportional hazards model for the effect of RBC count on outcome events in patients with HFmrEF.

	Male (*N* = 1095)		Female (*N* = 596)		Total (*N* = 1691)	
	Hazard ratio (95% CI)	*p* value	Hazard ratio (95% CI)	*p* value	Hazard ratio (95% CI)	*p* value
**Ⅰ. Adjust I: Cardiovascular death**						
**A**. RBC count as a continuous variable	0.63 (0.53, 0.75)	**<.0001**	0.51(0.40, 0.67)	**<.0001**	0.59 (0.51, 0.67)	**<.0001**
**B**. RBC count as a categorical variable						
Male RBC count ≥4.0 × 10^12^/L or Female RBC count ≥3.5 × 10^12^/L	(Reference)		(Reference)		(Reference)	
Male RBC count <4.0 × 10^12^/L or Female RBC count＜3.5 × 10^12^/L	1.86 (1.43, 2.40)	**<.0001**	2.11 (1.50, 2.97)	**<.0001**	1.97 (1.61, 2.42)	**<.0001**
**Ⅱ. Adjust II: Cardiovascular death**						
**A**. RBC count as a continuous variable	0.77 (0.63, 0.94)	**.0111**	0.57 (0.42, 0.77)	**.0002**	0.70 (0.59, 0.82)	**<.0001**
**B**. RBC count as a categorical variable						
Male RBC count ≥4.0 × 10^12^/L or Female RBC count ≥3.5 × 10^12^/L	(Reference)		(Reference)		(Reference)	
Male RBC count <4.0 × 10^12^/L or Female RBC count <3.5 × 10^12^/L	1.42 (1.07, 1.89)	**.0168**	1.79 (1.20, 2.67)	**.0043**	1.50 (1.19, 1.88)	**.0005**
**Ⅲ. Adjust I: Cardiovascular event**						
**A**. RBC count as a continuous variable	0.91 (0.82, 1.02)	.1149	0.98 (0.83, 1.16)	.8215	0.93 (0.85, 1.02)	.1421
**B**. RBC count as a categorical variable						
Male RBC count ≥4.0 × 10^12^/L or Female RBC count ≥3.5 × 10^12^/L	(Reference)		(Reference)		(Reference)	
Male RBC count <4.0 × 10^12^/L or Female RBC count <3.5 × 10^12^/L	1.16 (0.98, 1.38)	.0802	0.98 (0.77, 1.24)	.8477	1.10 (0.96, 1.26)	.1863
**Ⅳ. Adjust II: Cardiovascular event**						
**A**. RBC count as a continuous variable	0.89 (0.79, 1.01)	.0784	0.98 (0.81, 1.19)	.8656	0.91 (0.82, 1.01)	.0739
**B**. RBC count as a categorical variable						
Male RBC count ≥4.0 × 10^12^/L or Female RBC count ≥3.5 × 10^12^/L	(Reference)		(Reference)		(Reference)	
Male RBC count <4.0 × 10^12^/L or Female RBC count <3.5 × 10^12^/L	1.19 (0.99, 1.43)	.0692	0.91 (0.70, 1.19)	.4997	1.09 (0.94, 1.27)	.2451

*Note*: *The population was classified according to whether the male RBC count was ≥4.0 × 10^12^/L and whether the female RBC count was ≥3.5 × 10^12^/L. Bold represent significant values (*p* < .05).

Adjust I, adjusted for age and body mass index.

Adjust II, adjusted for age, body mass index, coronary heart disease, hypertension, hyperlipidemia, atrial fibrillation, diabetes mellitus, previous stroke, previous myocardial infarction, percutaneous coronary intervention, chronic obstructive pulmoriary disease, renal insufficiency, New York Heart Association functional class, uric acid, estimated glomerular filtration rate, potassium, left‐ventricular ejection fraction, current smoker and current drinker at baseline.

Abbreviations: CI, confidence interval; HFmrEF, heart failure with mildly reduced left ventricular ejection fraction; RBC, red blood cell.

**Figure 2:**
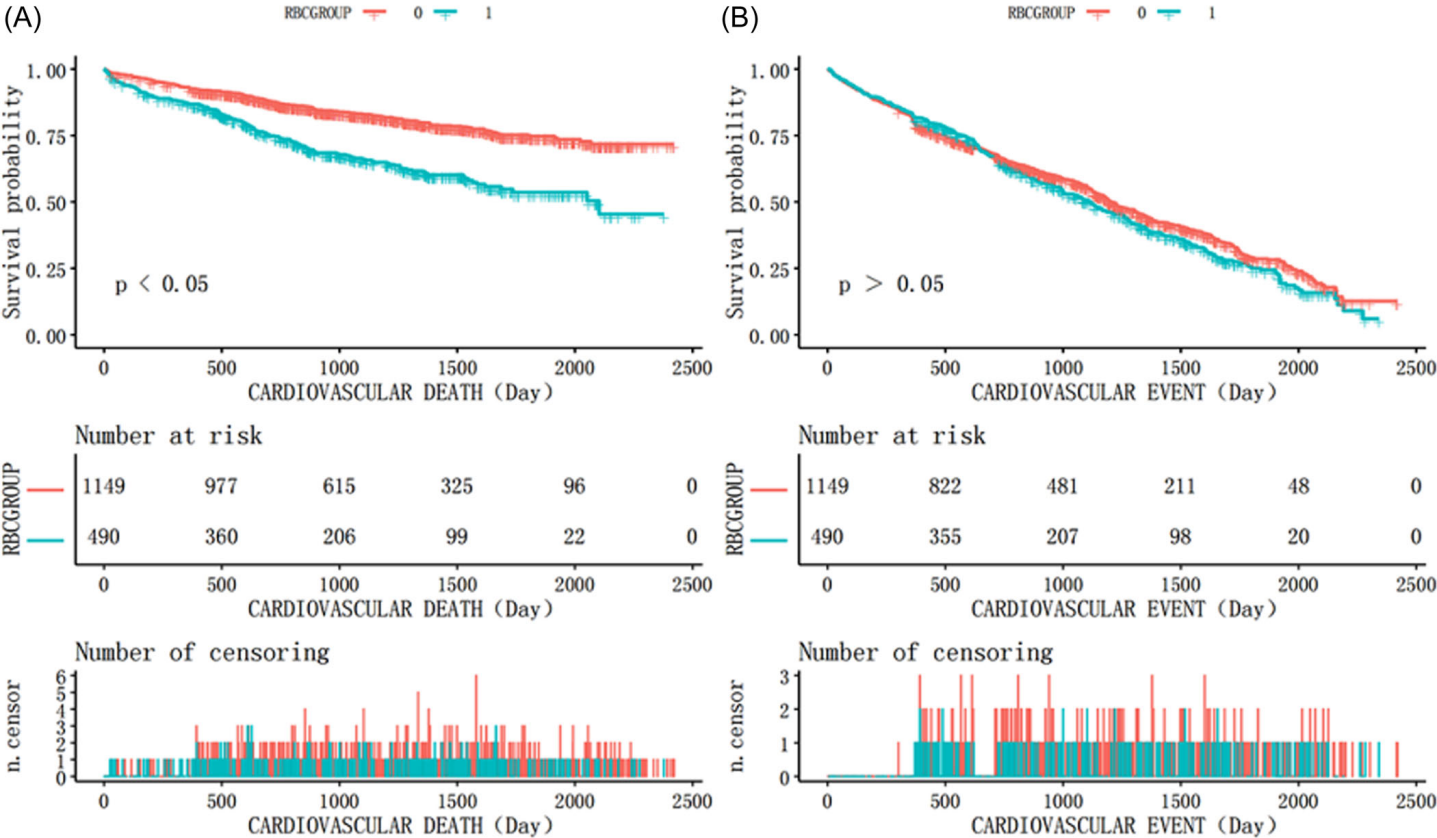
Kaplan–Meier curves grouped according to whether or not RBC decreases (0 = normal RBC group，1 = reduced RBC group). (A) Cumulative cardiovascular death. (B) Cumulative cardiovascular events. RBC, red blood cell.

After adjusting for all covariates, each 1.0 × 10^12^ cell/L increase in the RBC count was associated with a 30% lower risk of overall CV death (HR: 0.70; 95% CI: 0.59–0.82; *p* < .0001). The risk of CV death was reduced by 43% in women (HR: 0.57; 95% CI: 0.42–0.77; *p* = .0002) and by 28% in men (HR: 0.77; 95% CI: 0.63–0.94; *p* = .0111) (Table 3, Model II[A]). Patients with reduced RBC counts had a 50% higher risk of CV death than patients with normal RBC counts (HR: 1.50; 95% CI: 1.19–1.88; *p* = .0005), including a 79% higher risk in women (HR: 1.79; 95% CI: 1.20–2.67; *p* = .0043) and a 42% higher risk in men (HR: 1.42; 95% CI: 1.07–1.89; *p* = .0168) (Table 3, Model II[B]). Kaplan–Meier survival curves also demonstrated that patients with reduced RBC counts had a higher risk of CV death than patients with normal RBC counts (Figure 2A). However, the RBC count was not significantly correlated with the CV event rate (Figure 2B and Table 3, Model IV[A,B]).

Table S1 shows the patient profiles before and after propensity score matching. Age, body mass index, smoking status, alcohol use, hypertension, hyperlipidemia, diabetes, myocardial infarction, percutaneous coronary intervention, chronic obstructive pulmonary disease, renal insufficiency, New York Heart Association functional class, uric acid concentration, estimated glomerular filtration rate and potassium concentration differed between the two groups before propensity score matching (*p* < .05). The two groups had similar gender, coronary heart disease, atrial fibrillation, previous stroke, and LVEF (*p* > .05). After propensity score matching, both groups were well‐matched in terms of parameters. All *p* values had been greater than .05, indicating no difference between the two groups, and were comparable. Table S2 describes the propensity score‐matched cohort's primary and secondary outcome risk. Propensity score matching yielded results similar to the Cox proportional risk regression model. Patients in the group with reduced RBC counts had 46% higher cardiovascular mortality than those in the group without reduced RBC counts (HR: 1.46; 95% CI: 1.07–1.99; *p* = .018). However, the incidence of CV events was not significantly correlated between the two groups (HR: 1.10; 95% CI: 0.83–1.47; *p* = .506).

Table S3 outlines the results from a multivariate Cox proportional hazards model, elucidating the impact of Hemoglobin and RBCSV on cardiovascular mortality in patients with HFmrEF. The non‐adjusted, Adjust I and Adjust II models consistently present statistically significant associations. Precisely, each unit increase in Hemoglobin corresponded to a HR of 0.98 in both the non‐adjusted and Adjust I model for all genders (*p* < .0001) and 0.99 in the Adjust II model (*p* = .0004 for males *p* < .0001 for females). Similarly, each unit increase in RBCSV yielded an HR of 0.94 for males and 0.96 for females in the non‐adjusted model (*p* < .0001 for males, *p* = .0002 for females), 0.96 for males and 0.94 for females in the Adjust I model (*p* < .0001 for both), and 0.97 for males and 0.95 for females in the Adjust II model (*p* = .0033 for males, *p* = .0006 for females). These findings underscore the clinical significance of Hemoglobin and RBCSV as potential indicators for prognosis and management in patients with HFmrEF.

## 4 DISCUSSION

This study showed that reduced RBC counts were associated with increased CV mortality in patients with HFmrEF after adjusting for covariates. However, there was little correlation between changes in RBC counts and the incidence of CV events in patients with HFmrEF.

Anemia is common in patients with HFrEF or HFpEF and is associated with a poorer long‐term prognosis.^4^, ^5^, ^6^, ^8^, ^9^, ^10^ In a meta‐analysis involving more than 150 000 patients with HF from 33 studies, anemia doubled the risk of death.^11^ Compared with patients without anemia, those with anemia are older, more likely to be female, and more likely to have diabetes, chronic kidney disease (CKD), and severe HF with a worse functional status.^4^, ^6^, ^12^, ^13^ In addition, patients with HF and anemia have several comorbidities, including CKD, malnutrition associated with cardiac cachexia, and a low albumin concentration, all of which may worsen the prognosis.^9^, ^12^, ^14^

Furthermore, anemia and CKD often coexist in patients with HF. One study showed that although anemia doubled the risk of death in patients with HF, the adjusted risk of death was further increased by 1.5‐fold in the presence of CKD.^14^ The above‐mentioned studies and ours have demonstrated the association between HF and anemia. However, the difference is that unlike our study, previous studies failed to focus specifically on patients with HFmrEF. Our study demonstrates a relationship between RBC count and prognosis in patients with HFmrEF.

Severe anemia may progress to HF syndrome, which disappears when the anemia is corrected.^15^ The erythropoietin concentration increases in proportion to the severity of HF.^13^, ^16^ The relationship between renal blood flow and erythropoietin secretion in patients with HF is complex and not fully understood.^17^ For many years, correction of anemia in patients with HF was thought to improve symptoms, quality of life, and clinical outcomes. However, research has shown that treating anemia with erythropoiesis‐stimulating agents does not improve clinical outcomes but is associated with higher rates of thromboembolic events.^3^ Correction of anemia with erythropoiesis‐stimulating agents in patients with CKD with or without HF is associated with increased adverse events (including death).^18^, ^19^, ^20^, ^21^, ^22^ Iron deficiency is also a significant cause of anemia. A meta‐analysis of randomized controlled trials comparing ferric carboxymaltose versus placebo showed that ferric carboxymaltose administration was associated with reduced mortality and HF hospitalization in iron‐deficient patients with HFrEF.^23^ Although the clinical benefit of intravenous iron in anemic patients with HF has been demonstrated, the long‐term (>1 year) clinical benefit is uncertain.^24^

Furthermore, studies have also shown that an increased hemoglobin concentration in patients with HFrEF increases systemic vascular resistance and decreases the LVEF.^6^, ^9^, ^13^ These findings may also explain why some patients with HF exhibited correction of anemia but not a reduction in adverse outcome events. Some patients who have HF with anemia can experience a spontaneous increase in hemoglobin, and the prognosis of these patients is similar to that of patients who have HF without anemia.^25^ No studies have focused on the correction of anemia in patients with HFmrEF. Therefore, whether using drugs to correct anemia in patients with HFmrEF will lead to adverse outcomes remains unclear.

### 4.1 Limitations

This study had several limitations. First, this was designed as a retrospective study to minimize bias in patient selection; however, unobserved confounders remained. Second, we exclusively recruited patients from an isolated population at a local heart center in China, and the study population thereby lacked adequate diversity to justify the uniformity of the findings. Finally, because of the study's retrospective nature, some test results could not collected; the most important of these was the absence of data on the B‐type natriuretic peptide concentration.

## 5 CONCLUSIONS

Our findings suggest that reduced RBC counts are associated with increased CV mortality in patients with HFmrEF. Women with HFmrEF are more sensitive to changes in RBC counts than are men. Compared with men with HFmrEF, women with reduced RBC counts had a greater risk of CV death, and an increased RBC count was associated with a more significant reduction in the risk of CV death in women. Correcting a decreased RBC count can potentially reduce the risk of CV death in patients with HFmrEF. However, there is no clear conclusion regarding the need for medication to correct anemia in patients with HFmrEF. Further research is needed to understand the risk associated with anemia in patients with HFmrEF. A better understanding of risk factors for anemia in patients with HFmrEF may help to develop strategies to improve outcomes of patients with this severe disease.

## AUTHOR CONTRIBUTIONS

**Zhican Liu, Yunlong Zhu, and Lingling Zhang**: main authors of the study, established the idea to study the heart failure with mildly reduced ejection fraction in Chinese population. Writing main ideas for this research, main results and discussion of the findings. **Zhican Liu**: was a major contributor in writing the manuscript. **Mingxin Wu, Haobo Huang, Ke Peng, and Wenjiao Zhao**: interpreted statistical analysis and conducted multivariate analysis to prove the main findings of this project. S**ihao Chen, Xin Peng, Na Li, Hui Zhang, Yuying Zhou, Jie Fan and Yiqun Peng**: data collected and follow‐up. Jianping Zeng: corresponding author of the study, contributed on editing this manuscript and giving advice for the main authors to organise the manuscript and ideas of the project.

## CONFLICT OF INTEREST STATEMENT

The authors declare no conflicts of interest.

## Supporting information

Supporting information.

## Data Availability

The datasets generated and analyzed during the current study are not publicly available due the database owner is reluctant to make them public but are available from the corresponding author upon reasonable request.
