# Supplementary material for: Red blood cell count and risk of adverse outcomes in patients with mildly reduced left ventricular ejection fraction
Source: Clin Cardiol. 2023 Aug 4;46(10):1276–84. doi: 10.1002/clc.24108 (PMC10577554; doi:10.1002/clc.24108)
Supplement: Supplementary file 1 — Supporting information. [file CLC-46-1276-s001.docx]

| **Table S1** **Baseline Characteristics before and after Propensity-Score Matching** | | | | | | |
| --- | --- | --- | --- | --- | --- | --- |
| Characteristic | Before propensity matching | | | After propensity matching | | |
|  | Group A | Group B | *P-value* | Group A | Group B | *P value* |
| No | N=1149 | N=490 |  | N=389 | N=389 |  |
| Age, years | 67.6 ± 12.1 | 70.4 ± 12.5 | **<0.001** | 70.37 ± 12.0 | 71.08 ± 12.1 | 0.4138 |
| Male, N (%) | 732 (63.7%) | 335 (68.4%) | 0.07 | 262 (67.4) | 263 (67.6) | 1 |
| Body mass index, kg/m2 | 25.3 ± 4.1 | 24.6 ± 4.1 | **<0.001** | 24.87 ± 3.9 | 24.87 ± 4.1 | 0.9857 |
| Current smoker, N (%) | 392 (34.1%) | 139 (28.4%) | **0.023** | 111 (28.5) | 117 (30.1) | 0.6937 |
| Current drinker, N (%) | 116 (10.1%) | 27 (5.5%) | **0.003** | 18 (4.6) | 24 (6.2) | 0.4276 |
| Coronary heart disease, N (%) | 909 (79.1%) | 377 (76.9%) | 0.327 | 297 (76.3) | 308 (79.2) | 0.3886 |
| Hypertension, N (%) | 762 (66.3%) | 368 (75.1%) | **<0.001** | 271 (69.7) | 281 (72.2) | 0.4772 |
| Hyperlipidemia, N (%) | 259 (22.5%) | 78 (15.9%) | **0.002** | 65 (16.7) | 67 (17.2) | 0.9239 |
| Atrial fibrillation, N (%) | 208 (18.1%) | 74 (15.1%) | 0.141 | 75 (19.3) | 69 (17.7) | 0.6444 |
| Diabetes, N (%) | 347 (30.2%) | 189 (38.6%) | **<0.001** | 130 (33.4) | 134 (34.4) | 0.8203 |
| Previous stroke, N (%) | 145 (12.6%) | 60 (12.2%) | 0.834 | 50 (12.9) | 51 (13.1) | 1 |
| Myocardial infarction, N (%) | 634 (55.2%) | 215 (43.9%) | **<0.001** | 179 (46) | 191 (49.1) | 0.4297 |
| PCI, N (%) | 431 (37.5%) | 116 (23.7%) | **<0.001** | 100 (25.7) | 107 (27.5) | 0.6264 |
| COPD, N (%) | 131 (11.4%) | 75 (15.3%) | **0.029** | 56 (14.4) | 63 (16.2) | 0.5501 |
| Renal insufficiency, N (%) | 194 (16.9%) | 209 (42.7%) | **<0.001** | 125 (32.1) | 120 (30.8) | 0.7575 |
| NYHA, N (%) |  |  | **<0.001** |  |  | 0.5871 |
| Ⅱ | 520 (45.3%) | 175 (35.7%) |  | 153 (39.3) | 155 (39.8) |  |
| III | 413 (35.9%) | 185 (37.8%) |  | 151 (38.8) | 139 (35.7) |  |
| IV | 216 (18.8%) | 130 (26.5%) |  | 85 (21.9) | 95 (24.4) |  |
| Uric Acid,µmol/L | 354.8 ± 111.9 | 384.7 ± 125.8 | **<0.001** | 380.5 ± 116.7 | 378.3 ± 127.5 | 0.8019 |
| eGFR, ml/min/1.73m2 | 76.8 ± 31.8 | 51.2 ± 37.2 | **<0.001** | 62.22 ± 33.9 | 60.18 ± 36.2 | 0.4181 |
| Potassium, mmol/L | 4.1 ± 0.5 | 4.3 ± 0.7 | **<0.001** | 4.19 ± 0.6 | 4.20 ± 0.7 | 0.8165 |
| LVEF, % | 44.5 ± 2.7 | 44.4 ± 2.8 | 0.491 | 44.5 ± 2.7 | 44.4 ± 2.8 | 0.0639 |
| *The population was classified according to whether the male RBC count was ≥ 4.0 x 10^12^/L and whether the female RBC count was ≥ 3.5 x 10^12^/L. Values for continuous variables are given as means ±SD.Bold represent significant values (p < 0.05).Group A is RBC count ≥ 4.0 x 10^12^/L (Male) or RBC count ≥ 3.5 x 10^12^/L (Female); Group B is RBC count < 4.0 x 10^12^/L (Male) or RBC count < 3.5 x 10^12^/L (Female);  *Abbreviations: RBC:red blood cell;PCI:percutaneous coronary intervention;COPD :chronic obstructive pulmoriary disease;NYHA:New York Heart Association;eGFR:estimated glomerular filtration rate;LVEF:left ventricular ejection fraction. | | | | | | |

| **Table S2 Risk of Primary and Secondary Outcomes in the Propensity-Score–Matched Cohort** | | | | |
| --- | --- | --- | --- | --- |
| Outcome | No. of Patients with Event | Event Rate% | Hazard Ratio (95% CI) | *P Value* |
| Cardiovascular death |  |  |  | **0.018** |
| Group A | 98 | 25.2% | Reference |  |
| Group B | 128 | 32.9% | 1.46 (1.07, 1.99) |  |
| Cardiovascular event |  |  |  | 0.506 |
| Group A | 237 | 60.9% | Reference |  |
| Group B | 246 | 63.2% | 1.10 (0.83, 1.47) |  |
| *The propensity-score-matched cohort included 389 patients in the Group A and 389 patients in the Group B.Bold represent significant values (p < 0.05).Group A is RBC count ≥ 4.0 x 10^12^/L (Male) or RBC count ≥ 3.5 x 10^12^/L (Female); Group B is RBC count < 4.0 x 10^12^/L (Male) or RBC count < 3.5 x 10^12^/L (Female);  *Abbreviations: RBC:red blood cell ; | | | | |

| **Table S3 Results of a multivariate Cox proportional hazards model for the effect of Hemoglobin and Red blood cell specific volume on Cardiovascular death in patients with HFmrEF** | | | | | | |
| --- | --- | --- | --- | --- | --- | --- |
|  | Male | | Female | | Total | |
|  | hazard ratio (95% CI) | P value | hazard ratio (95% CI) | P value | hazard ratio (95% CI) | P value |
| Non-adjusted |  |  |  |  |  |  |
| Hemoglobin | 0.98 (0.98, 0.98) | **<0.0001** | 0.98 (0.97, 0.99) | **<0.0001** | 0.98 (0.98, 0.98) | **<0.0001** |
| Red blood cell specific volume | 0.94 (0.93, 0.96) | **<0.0001** | 0.96 (0.93, 0.98) | **0.0002** | 0.95 (0.93, 0.96) | **<0.0001** |
| Adjust I |  |  |  |  |  |  |
| Hemoglobin | 0.98 (0.98, 0.99) | **<0.0001** | 0.98 (0.97, 0.99) | **<0.0001** | 0.98 (0.98, 0.99) | **<0.0001** |
| Red blood cell specific volume | 0.96 (0.94, 0.97) | **<0.0001** | 0.94 (0.92, 0.97) | **<0.0001** | 0.95 (0.94, 0.96) | **<0.0001** |
| Adjust II |  |  |  |  |  |  |
| Hemoglobin | 0.99 (0.98, 0.99) | **0.0004** | 0.98 (0.97, 0.99) | **<0.0001** | 0.99 (0.98, 0.99) | **<0.0001** |
| Red blood cell specific volume | 0.97 (0.95, 0.99) | **0.0033** | 0.95 (0.93, 0.98) | **0.0006** | 0.96 (0.95, 0.98) | **<0.0001** |
| Bold represent significant values (p < 0.05) . Abbreviations: CI:confidence interval;HFmrEF:heart failure with mildly reduced left ventricular ejection fraction.  Non-adjusted model adjust for: None .  Adjust I, adjusted for age and body mass index.  Adjust II,adjusted for age, body mass index, coronary heart disease, hypertension, hyperlipidemia, atrial fibrillation, diabetes mellitus,  previous stroke, previous myocardial infarction, percutaneous coronary intervention, chronic obstructive pulmoriary disease, renal insufficiency, New York Heart Association functional class, uric acid, estimated glomerular filtration rate, potassium, left-ventricular ejection fraction, current smoker and current drinker at baseline. | | | | | | |
